# Molecular hydrogen as an adjuvant therapy may be associated with increased oxygen saturation and improved exercise tolerance in a COVID‐19 patient

**DOI:** 10.1002/ccr3.5039

**Published:** 2021-11-06

**Authors:** Ram B. Singh, Ghazi Halabi, Ghizal Fatima, Richa H. Rai, Alexander T. Tarnava, Tyler W. LeBaron

**Affiliations:** ^1^ Halberg Hospital and Research Institute Moradabad India; ^2^ Halberg Chrono‐Cardiology Center Aley Lebanon; ^3^ Era Medical College Lucknow India; ^4^ School of Physiotherapy Delhi Pharmaceutical Sciences and Research University Delhi India; ^5^ Drink HRW New Westminster British Columbia Canada; ^6^ Centre of Experimental Medicine Institute for Heart Research Slovak Academy of Sciences Faculty of Natural Sciences of Comenius University Bratislava Slovak Republic; ^7^ Molecular Hydrogen Institute Cedar City Utah USA; ^8^ Department of Kinesiology and Outdoor Recreation Southern Utah University Cedar Utah USA

**Keywords:** Antioxidant, free radical stress, hydrogen‐rich water, inflammation, post‐COVID‐19

## Abstract

Administration of molecular hydrogen dissolved in water to patient with COVID‐19‐like symptoms may improve oxygen levels and exercise capacity.

## Graphical abstract

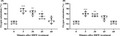

## 1 INTRODUCTION

COVID‐19 is associated with respiratory failure and subsequent hypoxia. New therapies that reduce hypoxia are urgently needed. Molecular hydrogen therapy may reduce inflammation, oxidative stress, and hypoxia. This case report suggests that hydrogen‐rich water may increase oxygen saturation and increase exercise tolerance in a patient with COVID‐19‐like symptoms.

Recent studies indicate that severe acute respiratory syndrome can develop from novel coronavirus (SARS‐CoV‐2), which may cause COVID‐19.^1^, ^2^ The clinical features of SARS‐CoV‐2 infection may range from silent infection to serious illness including death.^1^, ^2^ The disease is associated with pneumonia and consolidation in the lower and upper lungs that lead to respiratory failure with hypoxia.^1^, ^2^ Computerized tomography of the thorax may reveal a ground glass appearance of lungs, indicating viral pneumonia. There is an unmet need to develop new therapies for this disease, because COVID‐19 spreads rapidly, resulting in a pandemic with limited agents for treatment. In serious patients with COVID‐19, respiratory failure results in hypoxia, which is characterized by a lower oxygen saturation in the blood circulation.^1^, ^2^ Long term consequences of COVID‐19 include symptoms such as fatigue, headaches, joint pain, and dyspnea, as well as other neurological, lung, and heart damages.^3^ A recent WHO sponsored randomized trial following 11,330 adults across 30 countries and 405 hospitals found that the repurposed anti‐viral drugs remdesivir, hydroxychloroquine, interferon, and lopinavir or the combination of interferon and lopinavir had little to no impact on mortality.^4^ High doses of cortisone have been found to be useful among these patients.^5^ Mutations to the SARS‐CoV‐2 virus have created uncertainties regarding some treatment options, with recent research suggesting that monoclonal antibody therapies may be less effective against the emerging Delta variant.^6^

Recently, molecular hydrogen (H_2_) has been suggested for the treatment of COVID‐19, due to its potential antioxidant and anti‐inflammatory effects.^1^, ^7^, ^8^, ^9^ It is hypothesized that H_2_ therapy may decrease inflammation by decreasing the cytokine storm and by reducing free radical‐induced damage, which may decrease hypoxia and delay the need for oxygen therapy.^7^, ^8^, ^9^, ^10^, ^11^, ^12^ This hypothesis has led to the initiation of a large‐scale multicenter trial in Europe utilizing hydrogen‐producing tablets in high‐risk patients undergoing ambulatory care.^13^ The concept that molecular hydrogen may be a beneficial treatment for severe COVID‐19 is supported by a small trial in China that reported hydrogen inhalation was effective for reducing disease severity and dyspnea.^14^ Further, before undergoing this case report, the authors received private correspondence regarding the potential effectiveness of hydrogen‐producing tablets in a small, randomized placebo‐controlled trial with COVID‐19 patients (n = 24). Administration of four hydrogen‐producing tablets per day led to a significant reduction in interleukin‐6 (IL‐6) and fatigue (Sergej Ostojic MD, PhD, email correspondence May 7, 2021). Here, we report that molecular hydrogen, administered as hydrogen‐rich water (HRW), may increase oxygen saturation in the blood circulation and increase exercise tolerance.

## 2 CASE REPORT

### 2.1 Background

A male physician aged 77 years, presented with COVID‐19 like symptoms, potentially as a manifestation of side effects from the “Covishield” vaccination, developed by Oxford‐AstraZeneca, on April 17, 2021. Further details of this patient regarding clinical presentation and treatment given in acute stage have been reported earlier.^15^ After recovery from acute phase pneumonia consistent with COVID‐19 patients, this patient developed chronic hypoxia with oxygen saturation between 91% and 93%. In the first week of June 2021, (6 weeks after the acute phase of symptoms), his total and differential blood counts, as well as platelet counts, were within normal limits. His Hb level was 11.2 g/dl, aspartate aminotransferease (AST) 42.0 IU/L (normal up to 40 IU), and ferritin 305.60 ng/ml. His C‐Reactive protein (CRP) was 7.41 mg/L (normal, 0.0–6.0 mg/L), D Dimer 248.2 ng/ml (normal <500 ng/ml), and IL‐6, was 4.21 pg/ml (normal 0.0–0.5 pg/ml).

In view of the continuation of respiratory failure with hypoxia due to fibrosis of the lungs, his oxygen saturation at rest was 92%–93%. Four weeks post‐vaccination his echocardiogram revealed that his ejection fraction was >65%, and there was no evidence of cardiac dilatation. Breathing exercises with emphasis on abdominal breathing were the main stay of treatment. Nebuliser with asthalin + budicort + evolin were continuously and regularly administered twice daily. There was marked recovery after 6 weeks of treatment, but high‐resolution computerized tomography showed haziness in lower lung zones, indicating damage similar to post‐COVID‐19 fibrosis. Due to the presence of persistent fibrosis of the lungs, he was administered Nintedanib soft gelatin capsules 100 mg twice daily for 45 days, beginning 15 days after the onset of symptoms. He continued taking coenzyme Q10 (CoQ10) 200 mg twice daily, apixaban, and rosuvastatin 5 mg daily.

### 2.2 Hydrogen intervention

However, despite the treatment, there was still no improvement in oxygen saturation. Therefore, in addition to the above treatment, he was also administered HRW by using H_2_‐producing, magnesium‐based tablets (three tablets in the morning and two in the evening). Each tablet was dissolved in 200 ml of water, for a total daily intake of 1L of HRW. The hydrogen‐producing tablets used in this study are capable of delivering solutions well above saturation at standard ambient pressure and temperature, through quasi‐dissolved nanobubbles. The effective delivered hydrogen concentration was estimated at 8 mM (16 mg/L) based on extrapolation from available gas chromatography data on the hydrogen tablets obtained via H_2_ Analytics using gas chromatography (SRI 8610C), giving a total daily dose of ≈16 mg of molecular hydrogen per day. The effect of HRW treatment was assessed by measuring SpO_2_ levels before (0 min) and 5, 30, 45, and 60 min after treatment using the oximeter and multipara monitor. To assess the effects of combined exercise and HRW, the patient was advised physical training prior to HRW treatment. The exercise protocol was slow jogging for approximately two minutes and repeated three times with 3–5 min rest between. However, due to a marked decline in SpO_2_ levels of 81%–84%, the exercise could not be completed. Therefore, the exercise was done immediately after HRW treatment and SpO_2_ level was monitored during and after exercise at the same time intervals (5, 30, 45, and 60 min).

## 3 RESULTS

**FIGURE 1:**
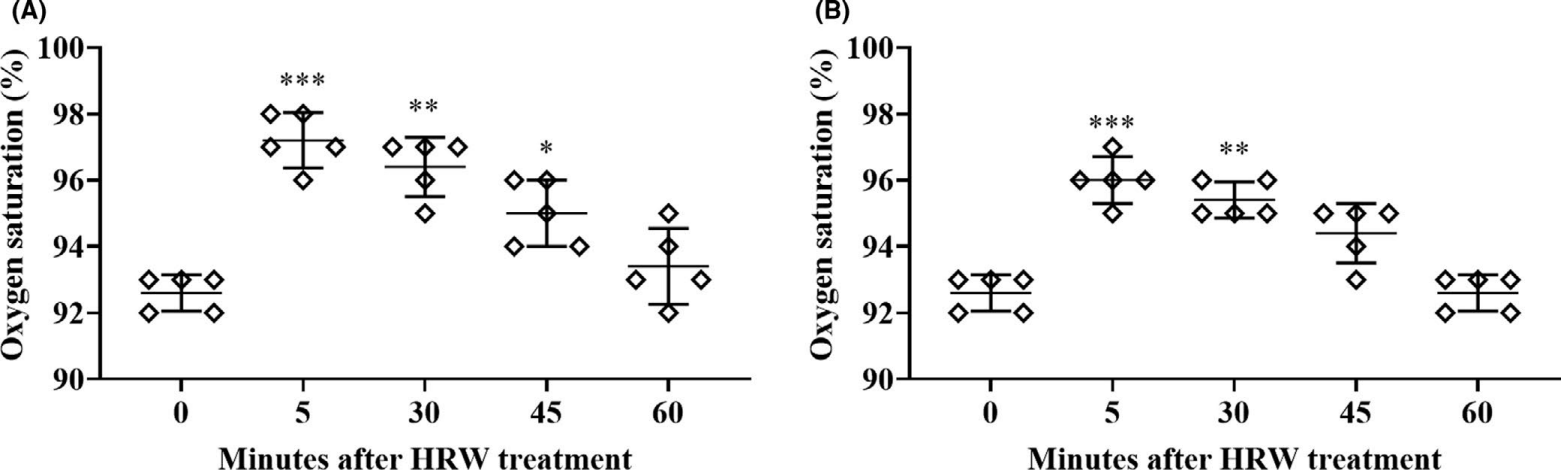
Mean oxygen saturation following five‐day treatment with HRW. (A) treatment with three H_2_‐producing tablets in the morning; (B) treatment with two H_2_‐producing tablets in the evening. Hydrogen‐producing tablets were dissolved in 200 mL of water each for a total daily intake of 1L of HRW. The results are presented graphically as mean ±SD with individual values for each daily measurement. *p < 0.05, **p < 0.01, ***p < 0.001 as compared to 0 (before treatment). Statistical differences were analyzed using repeated measure one‐way analysis of variance (ANOVA), followed by Tukey's Multiple Comparison Test using GraphPad Prism Software, ver. 8.0.2. (GraphPad Software).

Treatment with HRW was associated with significant changes in the oxygen saturation as measured via oximeter and multipara monitor (Figure 1). Five minutes after H_2_ treatment in the morning, mean oxygen saturation measured over the five‐day period increased from 92.6% to 97.2%, and then slowly decreased to 96.4%, 95%, and 93.4% after 30, 45, and 60 min, respectively (Figure 1A). Evening H_2_ treatment increased mean oxygen saturation from 92.6% to 96% after 5 min. After 30, 45, and 60, min mean oxygen saturation was 95.4%, 94.4%, and 92.6%, respectively (Figure 1B).

**FIGURE 2:**
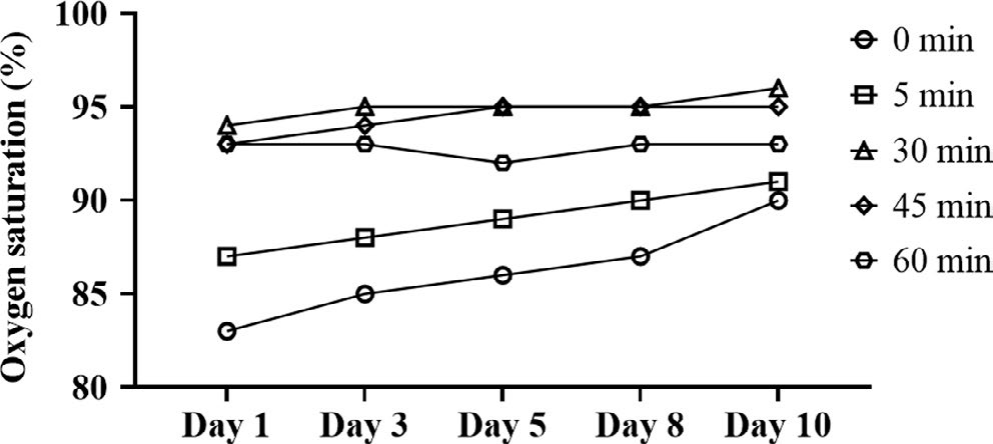
Time course of oxygen saturation after daily HRW treatment followed by exercise. The baseline SpO_2_ measurements (0 min) were performed just prior to HRW treatment

The effect of HRW treatment followed by exercise on oxygen saturation is presented in Figure 2. Regular HRW treatment with subsequent exercise increased basal oxygen saturation (measured before HRW administration) from 83% on day 1 to 90% on day 10. An additional marked increase in exercise tolerance was observed after 5, 30, 45, and 60 min on each day of the treatment, with the most prominent effect detected after 30 minutes (oxygen saturation ranged from 94% on day 1 to 96% on day 10).

## 4 DISCUSSION

This case report reveals that treatment with HRW was associated with a significant increase in oxygen saturation within 5 min of therapy in a patient with symptoms consistent with post‐acute COVID‐19‐like side effects such as subacute to chronic hypoxia (Figure 1). The findings also reveal that treatment with HRW may have increased exercise tolerance from hypoxia to near normal oxygen saturation (Figure 2).

Molecular hydrogen has been shown to protect the lungs from hypoxia/re‐oxygenation injury in mice by lowering free radical and inflammatory damages.^16^ Similar benefits were seen in a recent multicenter clinical trial utilizing hydrogen gas inhalation for chronic obstructive pulmonary disease (COPD).^17^ Hydrogen therapy improved breathlessness, cough, and sputum scale (BCSS) as compared to placebo. However, in this multicenter trial, there was no improvement in SpO_2_ in the hydrogen group as compared to the placebo group and change from baseline. Nevertheless, there were time by group interactions with respect to the changes from baseline in SpO_2_ (*p *< 0.0001).^17^ In data obtained via private correspondence, hydrogen water prepared via hydrogen‐producing magnesium tablets led to significantly lower fibrinogen levels (*p *= 0.03), prothrombin time (*p *= 0.02), and INR (*p *= 0.03) in COVID‐19 patients complicated with pneumonia, although this data set was from only five patients, so no concrete conclusions can be drawn (Sergej Ostojic MD, PhD, email correspondence May 7, 2021). Additionally, similarly prepared HRW demonstrated a significant improvement in VO_2_ and various exercise indices in middle‐aged overweight women after 28 days intake.^18^ Similarly, a lower hydrogen dosage of ~2 mg/day for 14 days also demonstrated improvements in peak oxygen uptake.^19^ Overall, hydrogen therapy has shown significant promise as an ergogenic agent for improved exercise performance and recovery.^20^ No study is available on the role of hydrogen showing increased oxygen saturation among patients with hypoxia or border line lower oxygen saturation, in COVID‐19 or from any other cause. Therefore, direct comparisons between this case report and the established literature are not possible.

Human studies have demonstrated that chronic consumption of hydrogen water may lower heart rate in subjects with metabolic syndrome (n = 60, 24 weeks),^21^ and acutely lower heart rate during strenuous exercise.^22^ Conversely another study reported that hydrogen water consumption has a heart rate raising effect in women observed at rest.^23^ Importantly, molecular hydrogen has been shown to potentiate the beneficial post‐infarct effects of hypoxic conditioning on rat hearts^24^, ^25^ and has shown to ameliorate the deleterious effects of intermittent hypoxia in rodent models.^25^, ^26^, ^27^, ^28^, ^29^

It seems that hydrogen therapy as HRW, may be an effective and novel adjuvant treatment against acute and post‐acute COVID‐19. Although the exact molecular mechanism of how hydrogen therapy provides benefits to COVID‐19 patients is not known, several mechanisms have previously been suggested.^7^, ^8^, ^9^, ^10^, ^11^, ^12^

Fatigue remains a common reported side effect of acute COVID‐19, with chronic fatigue being reported as one of the most common side effects of post‐acute COVID‐19.^30^ HRW as delivered by hydrogen‐producing magnesium tablets has shown to improve alertness equivalent to caffeine in a head‐to‐head crossover study in young, healthy, sleep‐deprived individuals (n = 23).^31^ Additionally, HRW was shown to result in a more robust stimulation of brain metabolism compared to caffeine, as measured via MRI, in a double‐blinded, placebo‐controlled RCT in young, healthy sleep‐deprived individuals.^32^ Molecular hydrogen was also shown to reduce both temporary and prolonged fatigue,^33^ suggesting it could be effective in eradicating post‐acute COVID‐19 weakness.

## 5 CONCLUSIONS

Treatment with HRW may enhance oxygen saturation in patients with hypoxia, as presented in this case study. Hydrogen therapy may also enhance exercise tolerance resulting in significantly lower episodes of hypoxia during physical training. It is proposed that hydrogen supplementation may produce selective anti‐oxidation, anti‐inflammation, anti‐apoptosis, favorable gene expression alterations, and act as a therapeutic gaseous‐signal modulator.

Hydrogen‐rich water may exert protective effects on individuals experiencing symptoms consistent with post‐acute COVID‐19, whether induced by viral infection or as a rare adverse event experienced by vaccination. Well‐controlled clinical trials are needed to confirm this preliminary case report.

## CONFLICTS OF INTEREST

AT is involved in commercial entities with interest in the marketing of hydrogen‐rich water. TWL has received travel reimbursement, honoraria, and speaking and consultancy fees from various academic and commercial entities regarding molecular hydrogen. All other authors report no conflict of interest.

## AUTHOR CONTRIBUTIONS

RBS, GH, GF, and RHR were involved in concept/design and data collection. RBS, ATT, and TWL wrote the article. All authors read and approved the final version of the manuscript.

## ETHICAL APPROVAL

This case report was conducted in accordance with the Declaration of Helsinki.

## CONSENT

Written and signed consent of the patient was obtained in accordance with journal's consent policy.

## Data Availability

All the data supporting the findings of this study are available within the article. No additional data is available.
